# N-Domain Isoform of Angiotensin I Converting Enzyme as a Marker of Hypertension: Populational Study

**DOI:** 10.1155/2012/581780

**Published:** 2012-05-15

**Authors:** Leila C. V. Maluf-Meiken, Fernanda B. Fernandes, Danielle S. Aragão, Fernanda A. Ronchi, Maria C. C. Andrade, Maria C. Franco, Andreia C. S. Febba, Frida L. Plavnik, José E. Krieger, Jose G. Mill, Ricardo C. C. Sesso, Dulce E. Casarini

**Affiliations:** ^1^Nephrology Division, Department of Medicine, Federal University of Sao Paulo, 740 Botucatu Street, 04023-900 Sao Paulo, SP, Brazil; ^2^Laboratory of Genetics and Molecular Cardiology, Heart Institute (InCor), Sao Paulo University Medical School, 05403-900 Sao Paulo, SP, Brazil; ^3^Department of Physiological Sciences, Federal University of Espirito Santo, 29075-910 Vitoria, ES, Brazil

## Abstract

The aim of this paper was to investigate the presence of the urinary 90 kDa N-domain ACE in a cohort of the population from Vitoria, Brazil, to verify its association with essential hypertension since this isoform could be a possible genetic marker of hypertension. Anthropometric, clinical, and laboratory parameters of the individuals were evaluated (*n* = 1150) and the blood pressure (BP) was measured. The study population was divided according to ACE isoforms in urine as follows: ACE 65/90/190, presence of three ACE isoforms (*n* = 795), ACE 90^+^ (65/90) (*n* = 186), and ACE 90^−^ (65/190) (*n* = 169) based on the presence (+) or absence (−) of the 90 kDa ACE isoform. The anthropometric parameters, lipid profile, serum levels of uric acid, glucose, and the systolic and diastolic BP were significantly greater in the ACE 90^+^ compared with the ACE 90^−^ and ACE 65/90/190 individuals. We found that 98% of individuals from the ACE 90^+^ group and 38% from the ACE 65/90/190 group had hypertension, compared to only 1% hypertensive individuals in the ACE 90^−^ group. There is a high presence of the 90 kDa N-domain ACE isoform (85%) in the studied population. The percentile of normotensive subjects with three isoforms was 62%. Our findings could contribute to the development of new efficient strategy to prevent and treat hypertension to avoid the development of cardiovascular disease.

## 1. Introduction

Essential hypertension (EH) is a multifactorial and polygenic disorder affecting around 27% of adults worldwide [1]. It has been considered a public health problem because of its major contribution to the global disease burden [2]. Hypertension is an important risk factor for cardiovascular diseases since it can lead to cardiac dysfunction and kidney and cerebrovascular diseases [1–4]. Despite the therapeutic agents available to treat this disease, blood pressure control in hypertensive patients is generally poor ranging from only 5% to 58% in different population studies [5]. Hypertension affects approximately 50 million individuals in the United States and 1 billion individuals worldwide [5, 6]. In Brazil, isolated studies have shown a high prevalence of the disease in the adult population, ranging from 22 to 44%, mostly above 25.0% [7]. 

Many pathophysiological factors have been implicated in the genesis of EH [4]. The renin-angiotensin system (RAS) accounts for the synthesis of several bioactive peptides playing a key role in the maintenance of blood pressure as well as fluid and salt balance homeostasis. Therefore, the RAS continues to attract the interest of many investigators exploring the role of genetic mechanism involved in the development of EH [8, 9]. Angiotensin converting enzyme (ACE) is a key component of the RAS by converting the inactive decapeptide angiotensin I (AngI) to angiotensin II (AngII), which is a potent vasoconstrictor [10], and by inactivating the vasodilator bradykinin [11]. ACE (EC 3.4.15.1) is a peptidyl-dipeptidase A [12] and there are two usually described isoforms: the somatic ACE (150–190 kDa) having two active sites C and N-domains, highly bounded to endothelial cells membrane, and the germinal ACE (90–110 kDa), found specifically in the testicles, exhibiting only a C-domain active site [13, 14]. N-domain soluble forms of ACE were described in human ileal fluid and in human and rats urine [15–21].

 Our group has recently described the 65 kDa N-domain isoform of ACE in the urine of both normotensive and hypertensive humans and rats, and the 90 kDa N-domain ACE isoform was solely found in the urine of hypertensive humans and rats [15–22]. According to these studies, the 90 kDa N-domain ACE was suggested as a genetic marker of hypertension [21]. These described N-domain enzymes are homologous isoforms to the N-terminal portion of the somatic ACE [19]. They are expressed in many different rat tissues and in mesangial cells of Wistar and Spontaneously Hypertensive Rats (SHRs) [19, 22–24] suggesting that these enzymes may influence the local production of AngII and modulate angiotensin (1–7) (Ang1–7) levels [19, 23, 24].

 Thus far, determinants of RAS activity are not completely known and it may play an important role in the pathogenesis of essential hypertension. Recently we described the association of 90 kDa N-domain ACE with plasma inflammatory markers, endothelial function, and family history of hypertension. Our data suggested that the 90 kDa ACE may be a marker for hypertension [25, 26]. Therefore, the aim of this study was to investigate the presence of the urinary 90 kDa N-domain ACE in a large cohort of the general population and to determine its association with presence of hypertension and with associated factors that can contribute to development of high blood pressure.

## 2. Methods

A cross-sectional study of cardiovascular risk factors was performed in the urban population of Vitoria, a city southeast region of Brazil, following the general guidelines of WHO-MONICA Project [27, 28]. A random sample of 1,661 households attended to the University Hospital to be submitted to clinical and laboratory exams to determine the cardiovascular risk profile. Subjects (age 25–64 years) were initially interviewed at their domiciles and then scheduled to a visit to the University Hospital where they were submitted to clinical and laboratory exams necessary to determine the cardiovascular risk. Details of recruitment and sample representation in the general population were described elsewhere [29].

Subjects attended the University Hospital in 12 h fasting to blood sample collection and further evaluation of height, weight, smoking habits, blood pressure, electrocardiogram, aortic pulse wave velocity, and use of antihypertensive drugs. Fasting glucose, cholesterol, triglycerides, and uric acid were determined by standard techniques in a central laboratory. Diabetes mellitus was defined as fasting glucose >125 mg/dL. During the domicile visit all individuals were orientated to collect all urine produced from 7 pm to 7 am to estimate electrolyte (Na, K) and creatinine nocturnal 12 h excretion. A sample of this urine was stored at −20°C and sent to a central laboratory to determine presence of ACE isoforms.

 Blood pressure (BP) was determined by trained nurses using standard mercury sphygmomanometer on the left arm after 5 min rest with the subject in the sitting position in three different times. The first and fifth phases of Korotkoff sounds were used for systolic and diastolic pressure, respectively. Clinic BP was determined as the mean value of two recordings obtained with a minimum 10 min interval. Presence of hypertension was defined as presence of clinic blood pressure ≥140/90 mmHg or use of antihypertensive drugs, including diuretics [30].

 This study was conducted in accordance with the Guidelines for Good Clinical Practice and the Declaration of Helsinki after approval by the Ethics Committees on Human Research from the Federal University of Espírito Santo (volume 4194/99-00) and from Federal University of Sao Paulo (0220/04). The informed consent was signed by all volunteers.

### 2.1. Urine Preparation and Western Blotting Analysis

Urine samples were collected with the addition of proteases inhibitors (complete TM, mini EDTA-free, Roche) and subsequent frozen. After unfrozen, pH was corrected to 8.0 with 1 mol/L TRIS buffer. These samples were centrifuged 2568 ×g and the supernatant was concentrated in Microcon (Millipore, USA). The protein concentration was determined by the Bradford method [31] (Bio-Rad Protein Assay Kit, Bio-Rad, USA) using bovine serum albumin as standard. After that, electrophoresis was performed on a 7.5% of slab gel in presence of SDS according to the Laemmli method [32] using 10 *μ*g denatured and reduced protein. Electrotransference was performed for 50 minutes at constant voltage (40 V) using a nitrocellulose membrane (Hybond ECL, GE Healthcare, Sweden). The membrane was incubated in a 5.0% nonfat dry milk blocking solution for 4 hours before overnight incubation at room temperature (20°C) with monoclonal antibody 9B9 (Chemicon International, USA) (diluted 1 : 1000). The subsequent steps were performed with the streptavidin/phosphatase alkaline system (GE Healthcare, Sweden) and the bands were revealed using substrates NBT/BCIP as recommended by the manufacturer (Bio-Rad, USA). In addition, the same researcher, blinded to clinical data, performed all western blotting of this survey.

### 2.2. DEAE-Cellulose Chromatography of Human Urine

To exemplify the urine separation of ACE isoforms we used a DEAE-cellulose chromatography procedure. The supernatant (100 mL) of dialyzed urine collected in presence of protease inhibitors (complete TM, mini EDTA-free, Roche) was chromatographed on a DEAE-cellulose cellex D column (1.5 × 10 cm) equilibrated with 20 mM Tris/HCl buffer, pH 7.0. Elution was carried out with a linear gradient of 20 mM to 500 mM Tris/HCl buffer, pH 7.0 at a flow rate of 60 mL/h, the protein elution profile was monitored by UV absorbance at 280 nm, and the fractions were assayed for ACE activity against Z-Phenyl-L-Histidyl-L-Leucine (Z-Phe-His-Leu) as substrate (Bachem Bioscience Inc) as described previously [19, 33].

### 2.3. Statistical Analysis

To evaluate the association between blood pressure levels, metabolic and anthropometric parameters, the study population was divided according to ACE isoforms in urine as follows: ACE 65/90/190 kDa, presence of three ACE isoforms; ACE 90^+^ (65/90 kDa), and ACE 90^−^ (65/190 kDa) based on the presence (+) or absence (−) of the 90 kDa N-domain ACE isoform. Based on our previous studies that the ACE 90^+^ group may be associated with higher BP levels [19], we established, prior to the data analysis, this group as the reference one for the comparisons with the other two groups. All continuous variables were examined for normality with the Kolmogorov-Smirnov test. The chi-square test was applied for the comparison of proportions. Analysis of variance followed by the Tukey test for pairwise comparisons was used to compare more than two independent means of continuous variables. Correlation between continuous variables was determined by Pearson's coefficient. Analysis of covariance was used to compare the mean values of blood pressure levels between ACE groups adjusting for potential confounding variables. Values of continuous variables are expressed as mean values ± SE. Statistical significance was set at *P* < 0.05.

## 3. Results

**Figure 1:**
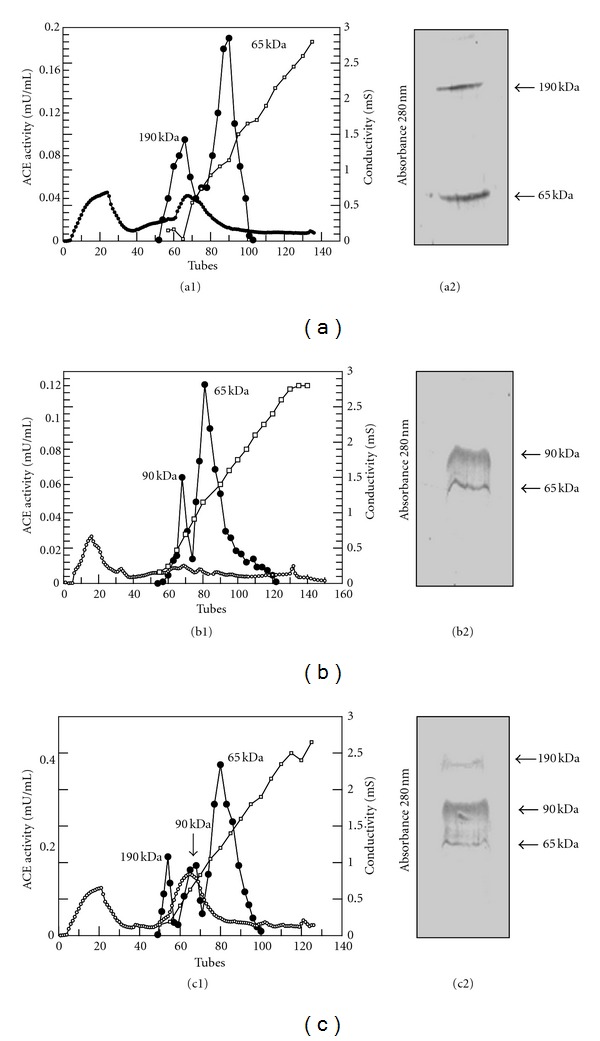
Chromatography of human urine from normal and hypertensive subjects on DEAE-cellulose. (a1) Normotensive subjects with two peaks with ACE activity corresponding to 190 and 65 kDa ACE; (b1) Hypertensive subjects with two peaks with ACE activity corresponding to 90 and 65 kDa ACE; (c1) Normotensive subjects with three peaks with ACE activity corresponding to 190, 90, and 65 kDa ACE. The dialyzed human urine (100 mL) was applied to a DEAE-cellulose column (1.5 × 10 cm). The column was washed with 20 mM Tris/HCl buffer, pH 7.0, and then eluted (fractions of 4.5 mL) with a linear gradient of 20 mM to 500 mM Tris/HCl buffer, pH 7.0, at a flow rate of 55 mL/h. (∘) Absorbance at 280 nm. (•) ACE activity with HHL as substrate. (□) Conductivity. Werstern blotting analysis of urinary ACEs. (a2) Normotensive subjects with 190 and 65 kDa ACE; (b2) Hypertensive subjects with 90 and 65 kDa ACE; (c2) Normotensive subjects with 190, 90, and 65 kDa ACE (as described in Section 2).

*3.1. ACE Isoforms and Activity Profile. *The study population was stratified according to ACE isoforms and the mean age of the overall population was 44 years, range 23–65 years. Profile of ACE isoforms found by Western blotting analysis using the antibody (9B9) is shown in Figure 1. After the Western blotting analysis, the urine prepared as described in Section 2 of three volunteers that presented with the 65, 90, and 190 kDa, 65 and 90 kDa, and 65 and 190 kDa ACE isoforms were submitted to a DEAE-cellulose chromatography. Profile of ACE isoforms in these three subjects are shown in Figure 1. The profile was obtained for urine of human subjects as described previously by our group [19, 25, 26].

### 3.1. Demographic, Anthropometric, and Clinical Characteristics

Demographic, anthropometric, and clinical characteristics of the sample stratified according to ACE isoforms are shown in Tables 1 and 2. Gender and ethnic distributions were significant different among the three ACE groups, whereas the smoking status and the proportion of diabetics were similar (Table 1). The anthropometric parameters, lipid profile, and serum levels of both uric acid and glucose were significantly greater in the ACE 90^+^ group compared with the ACE 90^−^ and ACE 65/90/190 groups (Table 2). In addition, individuals from the ACE 90^+^ group had higher urinary of sodium excretion when compared with the other groups. No difference was observed in potassium and creatinine excretion (Table 2).

 The mean systolic and diastolic BP was higher in the ACE 90^+^ compared to the ACE 90^−^ and the ACE 65/90/190 groups (Table 2). Since the hypertension was diagnosed according to presence of the blood pressure values higher than 140/90 mmHg in the clinic blood pressure measurement or the use of antihypertensive drugs, we also analyzed these groups separately.

**Figure 2:**
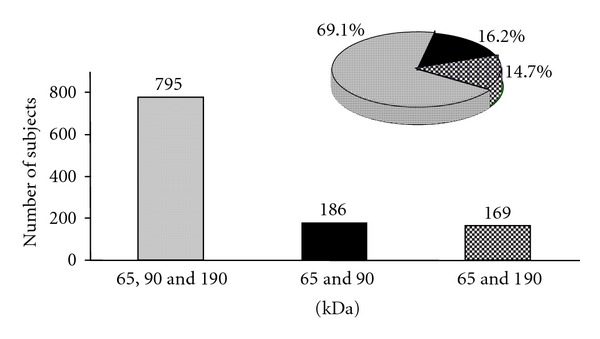
Distribution of ACE urinary isoforms and percentile of presence in subjects urine.

It was observed that 795 individuals presented with 65, 90, and 190 kDa, 186 individuals presented with 65 and 90 kDa, and 169 individuals presented with 65 and 190 kDa ACE isoforms in their urine (Figure 2).

For the entire cohort, significant positive associations were observed between blood pressure levels and age, BMI, waist-to-hip ratio, total cholesterol, triglycerides, uric acid, glucose, and urinary sodium excretion (Table 3). To establish if these correlations were not merely casual, multiple regression analyses were carried out. In a model adjusting for age, gender, race, BMI, waist-to-hip ratio, blood lipids, uric acid, sodium excretion, and glucose as independent variables, this analysis showed that age (*β* = 0.533; SE = 0.051; *P* < 0.001), BMI (*β* = 0.901; SE = 0.117; *P* < 0.001), serum uric acid (*β* = 1.513; SE = 0.377; *P* < 0.001), urinary sodium excretion (*β* = 0.024; SE = 0.008; *P* = 0.005), and glucose (*β* = 0.057; SE = 0.016; *P* = 0.008) were independent predictors of systolic blood pressure in the entire sample. Similar analysis was performed to diastolic blood pressure and we found that age (*β* = 0.164; SE = 0.033; *P* < 0.001), BMI (*β* = 0.685; SE = 0.077; *P* < 0.001), waist-to-hip ratio (*β* = 20.054; SE = 5.257; *P* < 0.001), uric acid (*β* = 0.706; SE = 0.446; *P* = 0.004), and urinary sodium excretion (*β* = 0.021; SE = 0.005; *P* = 0.005) reached statistical significance.

**Figure 3:**
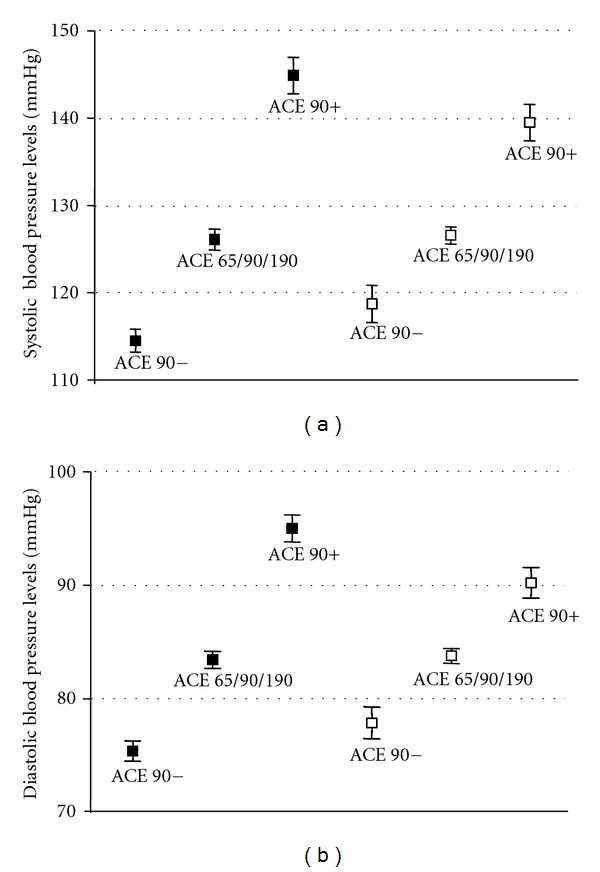
(a) Systolic and (b) diastolic blood pressure levels before (solid symbol) and after (open symbol) adjustment for age, gender, race, smoking status, diabetes incidence, antihypertensive use, BMI, waist-to-hip ratio, lipid profile, glucose, uric acid, and urinary sodium excretion. Values are mean ± SEM.

 Because multifactorial events are involved in the pathogenesis of hypertension, and high levels of blood pressure were found in individuals from the ACE 90^+^group, we performed an analysis of covariance controlling for potential confounding variables such as age, gender, race, smoking status, presence of diabetes, BMI, waist-to-hip ratio, antihypertensive drugs use lipid profile, glucose, uric acid and urinary sodium excretion. After these adjustments, the mean value for both diastolic and systolic blood pressure remained higher in the ACE 90^+^ group than that in the ACE 90^−^ and in the ACE 65/90/190 groups, although there was a attenuation in the blood pressure levels between groups (Table 4) (Figures 3(a) and 3(b)).

## 4. Discussion

 Different components of the RAS have been implicated in association with EH, a well-established cardiovascular risk factor affecting nearly 1 billion individuals worldwide [6]. Many studies show that the RAS exerts an important influence on water, sodium, and potassium homeostasis, thus influencing blood pressure regulation. ACE activity exerts a key role on the activity of the RAS and blood pressure regulation because this enzyme modulates angiotensin generation as well as bradykinin breakdown, a potent vasodilator peptide. ACE activity varies according to its isoforms. However, results of studies trying to associate functional variants of the RAS with EH have been contradictory [34–36].

 These contradictory findings may be explained for the majority of the studies by inadequate sample size leading to reduced statistical power. It is important to note that in the present study data were collected in a large and representative sample of the general population [37, 38].

 The sample studied had 1150 volunteers, 505 males and 645 females, 45.8 and 54.2%, respectively. According to the Brazilian 2000 Census, these percentages are close to the gender population distribution of Vitoria (46.2 and 53.8%) [39].

Urinary analysis of ACE isoforms showed that 85.3% of subjects present ACE 90 kDa isoform while in 14.7% of the subjects the 90 kDa ACE isoform was absent. Subjects presenting the three ACE isoforms 65/90/190 kDa represent the higher population subgroup (around 69.1%) and in this group 38% were hypertensive. These data strongly suggest that the presence of the 90 kDa N-domain ACE isoform in the urine could be associated with EH. These data confirm and extend previous studies of our laboratory where we detected a positive association of urinary 90 kDa ACE isoform with family history of hypertension and endothelial function in normotensive individuals [25]. We also described a direct association of 90 kDa N-domain ACE with plasma inflammatory markers and endothelial function [26]. A reduction in the basal NO production was suggested, confirmed by NO urine analysis in subjects with the 90 kDa N-domain ACE isoform alone or associated with a family history of hypertension. These data suggest that presence of the 90 kDa N-domain ACE itself may have a negative impact on flow-mediated dilatation stimulated by reactive hyperemia [26]. In addition, results of the studies of Ronchi et al. [22, 24] and Marques et al. [21] using normotensive and spontaneously hypertensive rats showed that this N-domain isoform could also be a possible genetic marker of hypertension.

The proteic profile of ACE isoforms found in the urine of volunteers repeated the previous results of our group [16–19, 25, 26], showing though that 90 kDa ACE isoform was present in urine of hypertensive subjects (42%) and was present in a very few percentage (1%) in urine of normotensive subjects (without 90 kDa ACE). The expression of the ACE with 90 kDa was detected in the group with the 65/90/190 ACE isoforms and in the group with 90 and 65 kDa. ACE activity profile using DEAE-cellulose chromatography was the same previously described by our group [19, 25, 26] for the aleatory samples analyzed.

We showed normotensive subjects with two peaks with ACE activity corresponding to 190 and 65 kDa ACE, hypertensive subjects with two peaks with ACE activity corresponding to 90 and 65 kDa ACE, and normotensive subjects with three peaks with ACE activity corresponding to 190, 90 and 65 kDa ACE. The analysis of expression by Werstern blotting of urinary ACEs showed that the bands corresponding to these peaks confirm the profile found in the chromatography.

Statistical analysis showed a positive association between 90 kDa N-domain ACE isoform and presence of hypertension. Since this finding was observed in a transversal study, we cannot establish a casual relationship between these variables. However we can speculate some casual relation between presence of 90 kDa isoform and age-dependent blood pressure increase because blood pressure values were quite low in the ACE 90^−^ group and hypertension was almost absent.

Several studies have shown that acid uric plasma level independently predicts hypertension development and can even be causal [40, 41]. It is considered a durable marker of risk for the development of EH [40]. We found a positive correlation of uric acid levels with both SBP and DBP through simple linear regression. To further evaluate the clinical relevance of this finding we evaluated the relations between these variables in the presence of risk factors. Through multivariate linear regression adjusted for age, gender, ethnicity, WHR, and lipid profile the association between both SBP and DBP with uric acid was still statistically significant, which evidences that this correlation persists even adjusted for other important hypertension-associated risk factors. These data are according to results of Teixeira et al. [25]; they described that subjects who presented with 90 kDa ACE had triglycerides levels higher than subjects without this isoform.

 In Pearsons's analysis we observed a positive correlation between age, BMI, WHR, cholesterol, triglyceride, glucose, uric acid, sodium excretion, and either SBP and DBP. In addition to covariance analysis in subjects with presence or absence of the 90 kDa N-domain ACE isoform, both associations were sustained for both SBP and DBP in the group with 90 kDa N-domain ACE isoform present.

Despite the correlation described previously, the physiological significance of the 90 kD ACE isoform remains to be determined. It will be important to determine whether it is a splicing variant, a posttranscription alteration product, or simply produced by the degradation of the 190 kDa isoform. Independent of the mechanism for production of the soluble form of N-domain ACE, this isoform may be physiologically and pathophysiologically important [42–46].

 Analysis of blood pressure levels could show that subjects with 90 kDa ACE have systolic and diastolic values higher than the group without this isoform. When the group with 90 and 65 isoforms was analyzed, they have systolic and diastolic levels higher than the other two groups (ACE 65/90/165 and ACE 90^−^). As shown in Figure 3 we can see that the early appearing of 90 kDa isoform either with three isoforms or with two isoforms seems to contribute to increase of diastolic and systolic levels. This can suggest that 90 kDa isoform can be involved in the increase of blood pressure, since ACE activity using specific substrates to N domain ACE isoform as ZPhe-His Leu was higher in hypertensive subjects. Data strongly suggest that normotensive subjects from group 1 (65, 90, and 190 kDa ACE isoforms) deserve special attention when it comes to prevention since 90 kDa N-domain isoform is positively associated with hypertension.

## 5. Conclusions

There is a high presence of the 90 kDa N-domain ACE isoform (85%) in the studied population. In addition this enzyme is associated with many known risk factors for cardiovascular disease. Although the mechanism involved in the generation of this isoform remains unknown, some physiological alterations can be associated with this expression in human urine and this should be used to assess hypertension risk in normotensive individuals. However, casual relationships should be established in longitudinal studies.

A second phase (5 years after) of this study is already being done prospectively analyzing ACE isoforms in the urine of these same volunteers, so causal associations may be evaluated. The percentile of normotensive subjects with three isoform was 62%; a prospective study of these subjects, specially, is very important to know how many will develop hypertension once they express the possible biomarker of hypertension (ACE 90 kDa) in their urine and will be possible confirm that this biomarker can really predict hypertension.

Our findings could contribute to the development of new efficient strategy to prevent and treat hypertension to avoid the development of cardiovascular disease.

The ACE 90 kDa absent is clearly associated with many important factors in maintenance of lower levels of blood pressure as (low weight, low salt ingestion, and low glucose tolerance) that increase with aging. These people can be a low proinflammatory status (opposite the group with the presence of the 90 kDa) and with low predisposition to increase of blood pressure levels. Finally the practical consequence of this study is that the 90 kDa ACE was associated with the presence of hypertension and the absence of this was associated with subjects with normal blood pressure levels, so this isoform would be a urinary marker of hypertension in normotensive subjects.

## Figures and Tables

**Table 1:** Demographic characteristics of the study population according to ACE isoforms.

Characteristics	ACE 90^+^	ACE 65/90/190	ACE 90^−^	*P* value
	*N* = 186	*N* = 795	*N* = 169	
Gender (%)				
Men	52	45	39	0.031
Women	48	55	61	
Race (%)				
White	34	33	37	
Black or Mulatto	61	60	50	0.002
Others	5	7	13	
Smoking status (%)				
Yes	24	25	27	0.850
No				
Diabetes mellitus (%)				
Yes	13	9	7	0.103
No				

**Table 2:** Anthropometric, clinical, and laboratory variables of the study population according to ACE isoforms.

Variables	ACE 90^+^	ACE 65/90/190	ACE 90^−^
	*N* = 186	*N* = 795	*N* = 169
Age (years)	48.5 ± 0.7*	43.9 ± 0.4	42.7 ± 0.8
BMI (kg/m^2^)	28.2 ± 0.4*	26.1 ± 0.2	25.2 ± 0.4
Waist-to-hip ratio	0.91 ± 0.01*	0.87 ± 0.03	0.85 ± 0.07
SBP (mmHg)	145.6 ± 1.4*	126.5 ± 0.8	114.9 ± 0.9
DBP (mmHg)	95.4 ± 0.8*	83.7 ± 0.5	75.7 ± 0.6
Cholesterol (mg/dL)	230.3 ± 3.6*	210.5 ± 1.6	208.4 ± 3.4
LDLc (mg/dL)	153.7 ± 3.3^#^	139.3 ± 1.5	140.4 ± 2.9
HDLc (mg/dL)	45.8 ± 1.0	45.7 ± 0.5	44.8 ± 0.8
Triglycerides (mg/dL)	162.2 ± 8.3*	134.4 ± 4.4	114.5 ± 7.1
Uric acid (mg/dL)	5.1 ± 0.1^#^	4.7 ± 0.1	4.4 ± 0.1
Glucose (mg/dL)	112.2 ± 3.2^#^	104.3 ± 1.1	100.6 ± 2.1
Urinary creatinine (mg/12 h)	700.0 ± 27.9	690.4 ± 12.6	683.2 ± 23.6
Urinary sodium (mEq/12 h)	108.8 ± 4.5^#^	97.8 ± 2.0	90.9 ± 3.9
Urinary potassium (mEq/12 h)	25.8 ± 1.5	23.2 ± 0.6	23.0 ± 1.0

Values expressed as mean ± SE. BMI: body mass index; SBP: systolic blood pressure; DBP: diastolic blood pressure; LDL: Low-density-lipoprotein cholesterol; HDL: High-density-lipoprotein cholesterol. *P < 0.001 and ^#^ *P* < 0.05 for the comparison of ACE 90^+^ versus ACE 90^−^ and ACE 90^+^ versus ACE 65/90/190 kDa groups.

**Table 3:** Pearson's correlation coefficient between blood pressure levels, anthropometric and clinical variables in the total sample.

	SBP	DBP	Age	BMI	WHR	Cholesterol	Triglycerides	Glucose	Uric acid
DBP	0.803**								
Age	0.351*	0.237*							
BMI	0.320*	0.335*	0.154*						
WHR	0.362*	0.399*	0.352*	0.408					
Cholesterol	0.187*	0.151*	0.304*	0.149*	0.137*				
Triglyceride	0.217*	0.262*	0.132*	0.202*	0.238*	0.347*			
Glucose	0.227*	0.145*	0.277*	0.251*	0.259*	0.215*	0.237*		
Uric acid	0.262*	0.288*	0.105	0.261*	0.396*	0.176*	0.342*	0.064	
Sodium excretion	0.141*	0.204*	0.041	0.177*	0.265*	0.015	0.084	0.108	0.119*

BMI: body mass index; WHR: Waist-to-hip Ratio; SBP: Systolic Blood Pressure; DBP: Diastolic Blood Pressure. *P < 0.05 and **P ≤ 0.001.

**Table 4:** Systolic and diastolic blood pressure levels and ACE isoforms in adjusted covariance analysis.

Groups	SBP (mmHg)	DBP (mmHg)
ACE 90^+^	138.4 (135.8 to 141.0)*	90.9 (89.2 to 92.6)*
ACE 65/90/190	127.0 (125.8 to 128.3)	84.1 (83.2 to 84.9)
ACE 90^−^	119.3 (116.5 to 122.1)	78.5 (76.7 to 80.4)

Values expressed as mean (95% confidence interval). SBP: Systolic Blood Pressure; DBP: Diastolic Blood Pressure. SBP and DBP are adjusted for age, gender, race, smoking status, diabetes incidence, antihypertensive drugs use, BMI, waist-to-hip ratio, lipid profile, glucose, uric acid, and urinary sodium excretion. *P < 0.001 for the comparison of ACE 90^+^ versus ACE 90^−^ and ACE 90^+^ versus ACE 65/90/190 kDa groups.
